# Dilated Coronary Sinus in Fetus: A Marker With Multiple Causes

**DOI:** 10.1016/j.jaccas.2026.108794

**Published:** 2026-06-17

**Authors:** Balaganesh Karmegaraj

**Affiliations:** Sowmi Pediatric Heart Centre, Tirunelveli & Department of Paediatric Cardiology, Amrita Institute of Medical Sciences and Research Centre, Kochi, India

**Keywords:** dilated coronary sinus, etiology, fetal echocardiography, fetus, 3D STIC imaging

**Figure:**
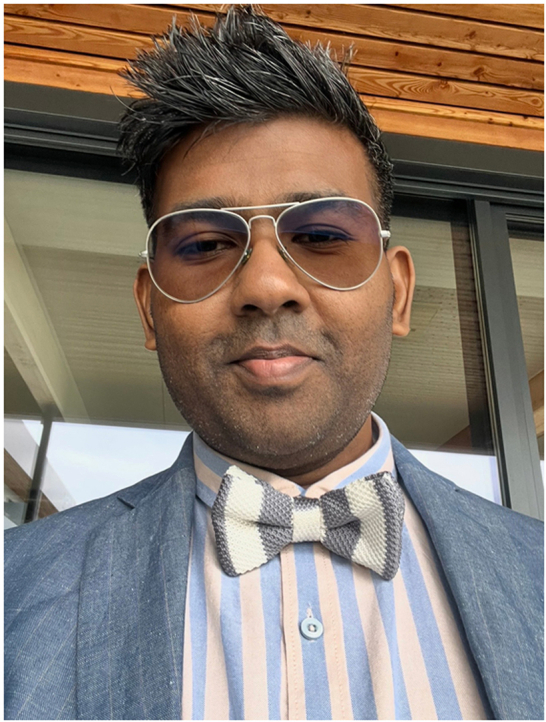

Dilated coronary sinus (CS) is a marker of a broad etiological spectrum. Bilateral superior vena cava (SVC) is the leading cause of CS dilation, usually without clinical significance unless combined with extracardiac or genetic anomalies. Although a dilated CS in a fetus is often benign, it is crucial to consider serious conditions such as total anomalous pulmonary venous connections. Hence it is important to assess for atypical pulmonary venous drainage into the CS and ensure persistent left SVC and right SVC drain properly into the right atrium. Rarely, abnormal SVC drainage into the left atrium can lead to cyanosis, stroke, or brain abscess.^1^^,^^2^ Some important causes for CS dilatation have been demonstrated in Figure 1, Figure 2, Figure 3, Figure 4, Figure 5 and summarized in the table in Figure 5C.^3^^,^^4^

**Figure 1:**
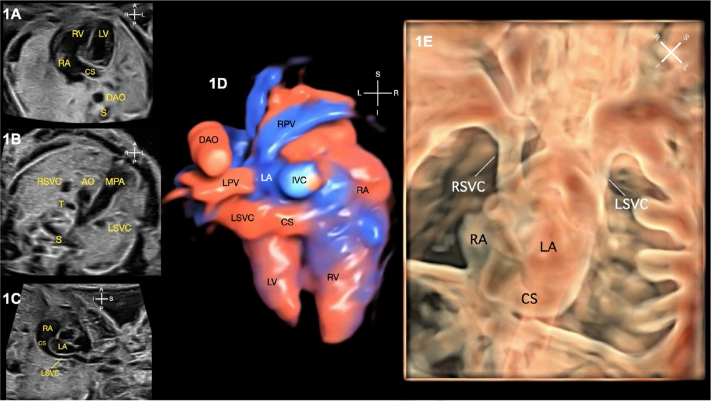
2-and 3-Dimensional Fetal Echocardiography of Bilateral Superior Vena Cava in a 24 Weeks Fetus (A to E) Bilateral SVC. (A) Four-chamber view showing dilated CS. (B) Four vessels in 3-vessel view. (C) Gray-scale image showing the entire LSVC drainage into the CS in sagittal view. (D and E) Four-dimensional STIC power Doppler (D) and HD live tissue surface rendering (E) images showing LSVC draining through CS into RA. AO = ascending aorta; CA = celiac axis; CS = coronary sinus; DAO = descending aorta; IVC = inferior vena cava; LA = left atrium; LPV = left pulmonary vein; LSVC = left superior vena cava; LV = left ventricle; MPA/PA = main pulmonary artery; RA = right atrium; RPV = right pulmonary vein; RSVC = right superior vena cava; RV = right ventricle; S = spine; STIC = spatiotemporal image correlation; SVC = superior vena cava; T = trachea.

**Figure 2:**
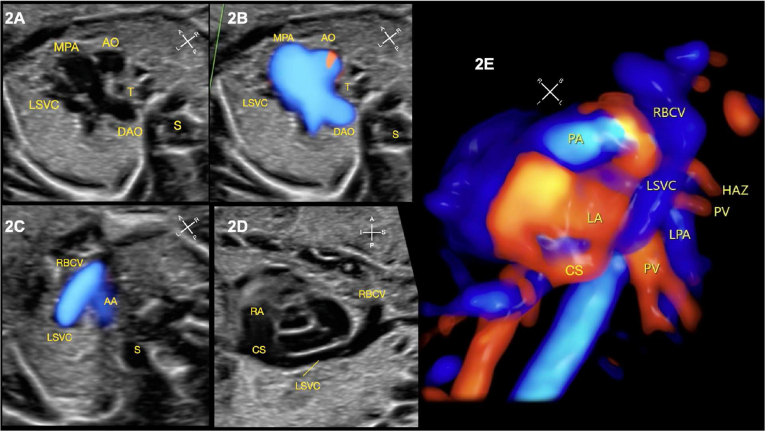
2-and 3-Dimensional Fetal Echocardiography of Isolated Left Superior Vena Cava in a 22 Weeks Fetus (A to E) Isolated LSVC. (A and B) Gray-scale (A) and power Doppler 3-vessel-tracheal (B) views showing abnormally oriented 3 vessels (absent RSVC and persistent LSVC). (C) Upper mediastinal view showing the RBCV. (D and E) Gray-scale (D) and 4-dimensional spatiotemporal image correlation STIC color Doppler (E) images showing the entire LSVC drainage into CS in sagittal view. Note the RBCV draining into LSVC. AA = aortic arch; HAZ = hemiazygos vein; RBCV = right brachiocephalic vein; other abbreviations as in Figure 1.

**Figure 3:**
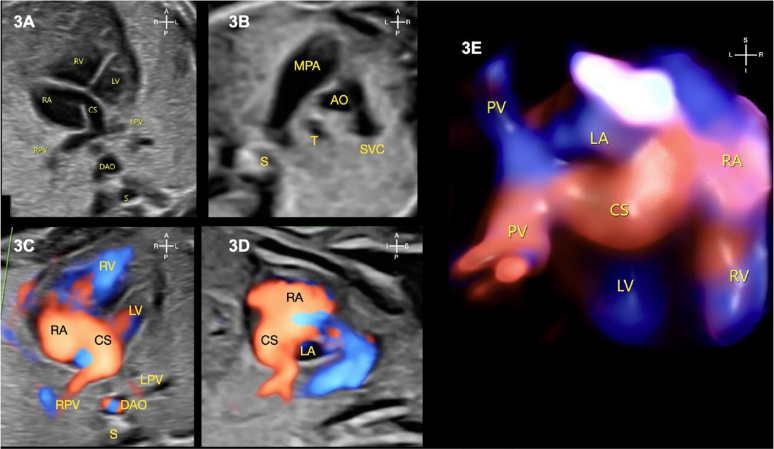
2-and 3-Dimensional Fetal Echocardiography of Coronary Sinus Total Anomalous Pulmonary Venous Connection in a 25 Weeks Fetus (A to E) CS TAPVC. (A) Four-chamber view showing dilated CS. (B) The 3-vessel view was normal. (C and D) Power Doppler 4-chamber (C) and sagittal (D) view showing the entire drainage of pulmonary veins into the CS. (E) STIC power Doppler posterior 4-chamber view showing the classic “whale tail sign” of CS TAPVC. TAPVC = total anomalous pulmonary venous connection; RPV = right pulmonary vein; other abbreviations as in Figures 1 and 2.

**Figure 4:**
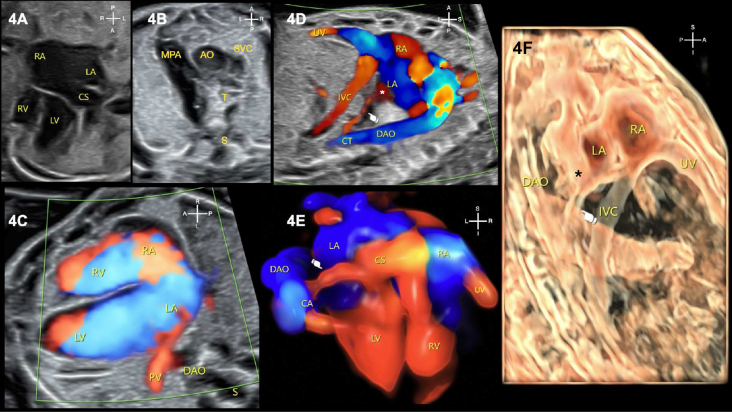
2-and 3-dimensional Fetal Echocardiography of Celiac Trunk Branch to Coronary Sinus Shunt With Umbilical Vein to Right Atrium Shunt in a 24 Weeks Fetus (A to F) CT branch–CS shunt with UV–RA shunt. (A) Four-chamber view showing dilated CS. (B) The 3-vessel view was normal. (C) Power Doppler 4-chamber view demonstrating normal pulmonary venous drainage. (D) Color Doppler sagittal view showing an anomalous branch from the CT draining into the CS. (E and F) Four-dimensional STIC power Doppler posterior 4-chamber view (E) and sagittal HD live tissue surface rendering showing the anomalous branch from the CT draining into the CS. ∗Coronary sinus. CT = celiac trunk; UV = umbilical vein; other abbreviations as in Figure 1, Figure 2, Figure 3.

**Figure 5:**
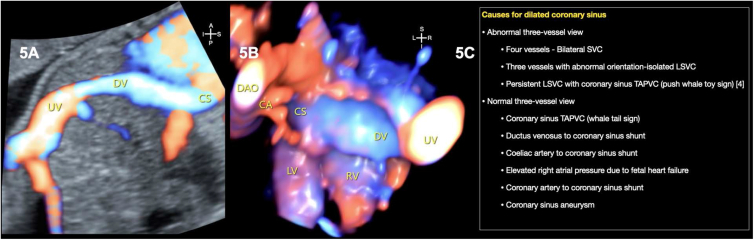
2-and 3-dimensional Fetal Echocardiography of Ductus Venous to Coronary Sinus shunt in a 34 Weeks Fetus and the Various Causes for Dilated Coronary sinus in Fetus (A and B) DV to CS shunt. (A) Power Doppler sagittal view of the fetal upper abdomen and lower thorax showing anomalous drainage of DV into the CS. (B) STIC power Doppler posterior 4-chamber view showing the anomalous drainage of DV into CS. CA = celiac axis; DV = ductus venosus; other abbreviations as in Figure 1, Figure 2, Figure 3, Figure 4.

In this issue of *JACC: Case Reports*, Torabyan et al^5^ described a rare case of CS aneurysm resulting in CS dilatation, with 22 months of postnatal follow-up. The authors noted that the CS aneurysm appeared benign, in contrast to ventricular outpouching.^5^ However, it is important to recognize that ventricular outpouching can occasionally demonstrate a benign course; therefore, these findings should be clearly communicated during parental counseling.^6^^,^^7^ The reported case involved atrioventricular re-entrant tachycardia with a concealed pathway. According to the literature, CS aneurysms or diverticula are well documented to possess accessory pathways. It has been proposed that the CS is surrounded by a myocardial coat, which establishes extensive connections with both the left and right atria. Accessory pathways arise from the connection between the myocardial coat—extending along the middle cardiac or posterior coronary vein and the ventricle.^8^^,^^9^ This case appears to be the first reported prenatal case featuring comprehensive prenatal and postnatal imaging. Otherwise, such cases are typically either asymptomatic or managed as supraventricular tachycardia in infancy, which can reappear in adulthood as recurrent supraventricular tachycardia or resemble a cardiac tumor or aneurysm.^10^^,^^11^ Recent developments in prenatal imaging have facilitated the early identification of lesions that were usually discovered incidentally during adulthood.^12^, ^13^, ^14^, ^15^ Although these advancements provide notable advantages, they also introduce complexities, including the potential for unwarranted termination of pregnancies in certain regions.

## Funding Support and Author Disclosures

The author has reported that he has no relationships relevant to the contents of this paper to disclose.

## References

[1] Karmegaraj B., Srimurugan B., Krishnan V., Vaidyanathan B. (2023). Clinical presentation, conventional/4D spatio-temporal image correlation imaging findings, pregnancy and early postnatal outcomes in fetuses having anomalies of systemic venous return in the absence of significant intracardiac defects: a retrospective study from two centres in Southern India. Echocardiography.

[2] Karmegaraj B. (2023). Whale's tail sign in fetus with coronary sinus total anomalous pulmonary venous connection. Ultrasound Obstet Gynecol.

[3] Zhang W.D., Ma B., Qi P.A. (2025). Anomalous drainage of the ductus venosus into the coronary sinus: prenatal ultrasound diagnosis utilizing two-dimensional and three-dimensional imaging techniques and differential diagnosis. BMC Pregnancy Childbirth.

[4] Karmegaraj B., Vijayakumar S., Krishnakumar R., Kottayil B. (Published online May 16, 2026). Prenatal diagnosis using 4D spatiotemporal image correlation echocardiography in two fetuses with atypical total anomalous pulmonary venous connection. Ultrasound Obstet Gynecol.

[5] Torabyan Z., Lindblade C., Nguyen M., Johnson R. (2026). Unique prenatal diagnosis of a coronary sinus aneurysm. JACC Case Rep.

[6] Karmegaraj B. (2023). Prenatal diagnosis, 4D spatiotemporal image correlation imaging, pregnancy, and postnatal outcome of partial Uhl's anomaly. Eur Heart J Cardiovasc Imaging.

[7] Zidere V., Gebb J., Vigneswaran T., Charakida M., Simpson J.M., Bower S. (2019). Spontaneous resolution of large pericardial effusion associated with right ventricular outpouching in four fetuses. Ultrasound Obstet Gynecol.

[8] Gerlis L.M., Davies M.J., Boyle R., Williams G., Scott H. (1985). Pre-excitation due to accessory sinoventricular connexions associated with coronary sinus aneurysms. A report of two cases. Br Heart J.

[9] Sun Y., Arruda M., Otomo K. (2002). Coronary sinus-ventricular accessory connections producing posteroseptal and left posterior accessory pathways: incidence and electrophysiological identification. Circulation.

[10] Hori S., Saito S., Kato T. (2021). Giant coronary sinus aneurysm misdiagnosed as an extracardiac mediastinal tumor. Gen Thorac Cardiovasc Surg.

[11] Bunck A.C., Eghbalzadeh K., Naehle C.P., Ten Freyhaus H., Maintz D., Wahlers T. (2018). Large saccular aneurysm at the ostium of the coronary sinus mimicking a right-atrial myxoma. Circ Cardiovasc Imaging.

[12] Karmegaraj B., Vijayakumar S. (2024). Prenatal diagnosis, conventional/4-dimensional imaging, pregnancy, and postnatal outcome of unicuspid unicommissural aortic valve. Circ Cardiovasc Imaging.

[13] Karmegaraj B., Vijayakumar S. (Published online February 22, 2026). Four-dimensional multiplanar imaging of isolated bileaflet pulmonary valve and pulmonary trunk aneurysm in fetus with TLL1 mutation. Ultrasound Obstet Gynecol.

[14] Karmegaraj B., Vijayakumar S. (Published online October 10, 2025). Isolated retroaortic left brachiocephalic vein imitating vertical vein: prenatal insights from 4D surface rendering. Ultrasound Obstet Gynecol.

[15] Karmegaraj B., Vijayakumar S. (2026). Prenatal diagnosis and postnatal outcomes of a rare venous anomaly: prominent azygos vein with an uninterrupted inferior vena cava. Am J Obstet Gynecol.

